# Genomic identification of a novel co-trimoxazole resistance genotype and its prevalence amongst *Streptococcus pneumoniae* in Malawi

**DOI:** 10.1093/jac/dkt384

**Published:** 2013-09-29

**Authors:** Jennifer E. Cornick, Simon R. Harris, Christopher M. Parry, Michael J. Moore, Chikondi Jassi, Arox Kamng'ona, Benard Kulohoma, Robert S. Heyderman, Stephen D. Bentley, Dean B. Everett

**Affiliations:** 1 Malawi-Liverpool-Wellcome Clinical Research Programme, University of Malawi, College of Medicine, Blantyre, Malawi; 2 Institute of Infection and Global Health, The University of Liverpool, Liverpool, UK; 3 Wellcome Trust Sanger Institute, Hinxton, UK; 4 Mahidol-Oxford Tropical Medicine Research Unit, Bangkok, Thailand; 5 Centre for Tropical Medicine, Nuffield Department of Clinical Medicine, University of Oxford, Oxford, UK; 6 Liverpool School of Tropical Medicine, Liverpool, UK

**Keywords:** trimethoprim, sulfamethoxazole, pneumococcal disease, prophylaxis

## Abstract

**Objectives:**

This study aimed to define the molecular basis of co-trimoxazole resistance in Malawian pneumococci under the dual selective pressure of widespread co-trimoxazole and sulfadoxine/pyrimethamine use.

**Methods:**

We measured the trimethoprim and sulfamethoxazole MICs and analysed *folA* and *folP* nucleotide and translated amino acid sequences for 143 pneumococci isolated from carriage and invasive disease in Malawi (2002–08).

**Results:**

Pneumococci were highly resistant to both trimethoprim and sulfamethoxazole (96%, 137/143). Sulfamethoxazole-resistant isolates showed a 3 or 6 bp insertion in the sulphonamide-binding site of *folP*. The trimethoprim-resistant isolates fell into three genotypic groups based on dihydrofolate reductase (encoded by *folA*) mutations: Ile-100-Leu (10%), the Ile-100-Leu substitution together with a residue 92 substitution (56%) and those with a novel uncharacterized resistance genotype (34%). The nucleotide sequence divergence and dN/dS of *folA* and *folP* remained stable from 2004 onwards.

**Conclusions:**

*S. pneumoniae* exhibit almost universal co-trimoxazole resistance *in vitro* and *in silico* that we believe is driven by extensive co-trimoxazole and sulfadoxine/pyrimethamine use. More than one-third of pneumococci employ a novel mechanism of co-trimoxazole resistance. Resistance has now reached a point of stabilizing evolution. The use of co-trimoxazole to prevent pneumococcal infection in HIV/AIDS patients in sub-Saharan Africa should be re-evaluated.

## Introduction

In sub-Saharan Africa, *Streptococcus pneumoniae* is one of the most common causes of meningitis and pneumonia. The combination of trimethoprim and sulfamethoxazole (co-trimoxazole) is recommended by WHO for prophylaxis in HIV/AIDS patients to prevent opportunistic bacterial infections and *Pneumocystis jirovecii.*^1^ Co-trimoxazole preventative therapy (CPT) became national policy in Malawi in 2005 and is now administered across all government-funded hospitals. In 2006, 34 942 Malawians were registered to receive CPT; by the end of 2010 this figure had increased to 189 520.^2^ Between 1993 and 2007, sulfadoxine/pyrimethamine was the recommended first-line treatment for uncomplicated malaria in Malawi and was used to prevent malaria in pregnancy.^3^ Prior to the introduction of CPT, co-trimoxazole resistance in *S. pneumoniae* in Malawi was already high at 74% and since 2005 resistance has been consistently above 90%.^4^

Co-trimoxazole and sulfadoxine/pyrimethamine both target dihydrofolate reductase (DHFR) and dihydropteroate synthase (DHPS), allowing cross-resistance mechanisms to these two antimicrobials.^5^ DHFR and DHPS form part of the folic acid biosynthetic pathway. Co-trimoxazole and sulfadoxine/pyrimethamine act as false substrate inhibitors, preventing folic acid biosynthesis and bacterial cell growth. Resistance to co-trimoxazole and sulfadoxine/pyrimethamine is conferred by acquisition of mutations in *folA* and *folP*, the genes encoding DHFR and DHPS, respectively. Although multiple mutations have been reported in trimethoprim-resistant pneumococci, the single substitution Ile-100-Leu in DHFR is sufficient to confer resistance.^6,7^ In sulfamethoxazole-resistant isolates, *folP* is characterized by a 3 or 6 bp insertion, resulting in the insertion of one or two amino acids in the DHPS sulphonamide-binding site.^8–10^

We analysed the *folA* and *folP* sequences of 143 pneumococci isolated from carriage and invasive disease following the introduction of CPT in Malawi, to define the molecular basis of co-trimoxazole resistance under the dual selective pressure of widespread co-trimoxazole and sulfadoxine/pyrimethamine use.

## Methods

### Ethics

We performed a detailed characterization of bacterial isolates from clinical specimens taken from patients for clinical reasons. The isolates used in the study were anonymized. These data are published with the approval of the University of Malawi College of Medicine Research & Ethics Committee and conform to institutional guidelines.

### Study isolates

The Malawi-Liverpool-Wellcome Trust Clinical Research Programme (MLW), based at Queen Elizabeth Central Hospital (QECH) in Blantyre, Malawi, has archived over 5000 pneumococcal isolates since 1996. A convenience sample of 143 pneumococci, collected between 2002 and 2008, was selected from the archive as part of a study into the genetic diversity of *S. pneumonia*e prior to the roll-out of 13-valent pneumococcal conjugate vaccine in Malawi.^11^ The selected isolates encompassed 35 serotypes and 68 multilocus sequence typing (MLST) sequence types (STs);^12^ 65 isolates belonged to one of 48 MLST STs that had not previously been reported to the MLST database and are here referred to as STunknown, followed by a number. One hundred and thirty-one pneumococci were from invasive disease (92% of the total) and 12 were from nasopharyngeal carriage (8%).

### Culture and extraction methodologies

Co-trimoxazole susceptibility was assessed using the BSAC disc diffusion method.^13^ Sulfamethoxazole and trimethoprim MICs were determined by the Etest (AB Biodisk, Solna, Sweden) according to the manufacturer's instructions. *S. pneumoniae* ATCC 49619 was used as a quality control strain and gave values within an acceptable reported range.

*S. pneumoniae* isolates that had been stored in Microbank bacterial preservers (Prolab Diagnostics, Ontario, Canada) were streaked onto blood agar plates and incubated at 37°C for 18 h. Single isolated colonies were suspended in Todd–Hewitt Broth (Oxoid, Basingstoke, UK) and incubated at 37°C for 18 h. The cells were sedimented by centrifugation and resuspended in 480μL of 50 μM EDTA and 120 μL of lysozyme and incubated at 37°C for 1 h. Genomic DNA was prepared from the lysate using a Promega Wizard Genomic DNA purification kit (Promega, Madison, USA). Multiplex DNA sequencing was performed with an Illumina Genome Analyzer GAII (Illumina, CA, USA), as described elsewhere.^14^ All of the sequence reads generated are deposited in the short read archive (National Centre for Biotechnology Information) under the accession numbers ERP000185 and ERP000152. Reads were assembled using Velvet v1.0.03,^15^ and contiguated against a complete reference sequence (accession number FM211187) using ABACUS.^16^ Serotype and MLST STs were determined as previously described.^17^ The *folA* and *folP* genes were identified by BLAST searching *folA_*R6 (Gene ID 4442919) and *folP_*R6 (Gene ID 4443057) from the fully susceptible laboratory reference strain *S. pneumoniae* R6 (trimethoprim MIC 2 mg/L; sulfamethoxazole MIC 16 mg/L). Significant hits were viewed, edited and annotated in Artemis V11.22.^18^ Nucleotide sequences of *folA* and *folP* were aligned separately in Seaview V4.1^19^ using Muscle V3.7.^20^ The nucleotide sequences were translated into amino acid sequences and a second alignment was performed.

The ratio of non-synonymous to synonymous single-nucleotide polymorphisms (dN/dS SNPs) in *folA* and *folP* for each isolate was computed using the Nei–Gojobori method.^21^

### Phylogeny construction

Phylogenetic trees were constructed using RAxML v.7.0.4^22^ using the nucleotide alignments. A generalized time-reversible model with gamma correction for among-site variation was used with 10 iterations using different starting trees. To assess support for relationships in the tree, 100 random bootstrap replicates were performed. *folA*_R6 or *folP*_R6 was included as an outgroup.

## Results

### In vitro resistance

Co-trimoxazole resistance amongst the study isolates was 96% (137/143). The MICs of sulfamethoxazole and trimethoprim for all resistant isolates were >1024 and >32 mg/L, respectively. This is very high-level resistance to both antimicrobials based on BSAC definitions.^13^ Six isolates were susceptible to co-trimoxazole. Five co-trimoxazole-susceptible isolates (53, 88, 96, 127 and 147) were susceptible to both trimethoprim and sulfamethoxazole (MICs 12 and 96 mg/L, respectively). The remaining co-trimoxazole-susceptible isolate, isolate 137, was susceptible to trimethoprim but resistant to sulfamethoxazole (MICs 6 and >1024 mg/L, respectively).

### Molecular markers of sulfamethoxazole resistance

All sulfamethoxazole-resistant isolates displayed an insertion of 3 or 6 bp in *fol*P, resulting in the insertion of one or two amino acids between Arg-58 and Ile-66 of DHPS, an area hypothesized to form the sulphonamide-binding site.^7^ The five sulphonamide-susceptible isolates did not display amino acid insertions in this area (Table 1). No additional mutations were detected that were unique to the sulphonamide-resistant isolates.

**Table 1.** Amino acid variation of DHPS from sulfamethoxazole-resistant pneumococci in relation to the co-trimoxazole-susceptible laboratory reference *S. pneumoniae* R6

*folP* insertion	DHPS mutation^a^	Number of isolates (%)	Previously reported in *S. pneumoniae*
Wild-type	—	5 (3)	^30^
6 bp	ST***RP***RPGSSYVEIE	88 (65)	^8,9,30^
3 bp	STRPG***R*_**SSYVEIE	21 (15)	^8^
6 bp	STRP***SSS***SSYVEIE	15 (10)	^30^
6 bp	STRPG***SY***SSYVEIE	2 (1)	^10^
6 bp	STRPG***TG***SSYVEIE	6 (4)	—
6 bp	STRPGSSY***VE***VEIE	2 (1)	—
6 bp	STRPGSSYV***EI***EIE	4 (3)	^8,10,31^

Amino acid insertions are in bold italics.

^a^Represents amino acid residues 56–67 in the amino acid sequence of DHPS from *S. pneumoniae* R6.

**Figure 1.**
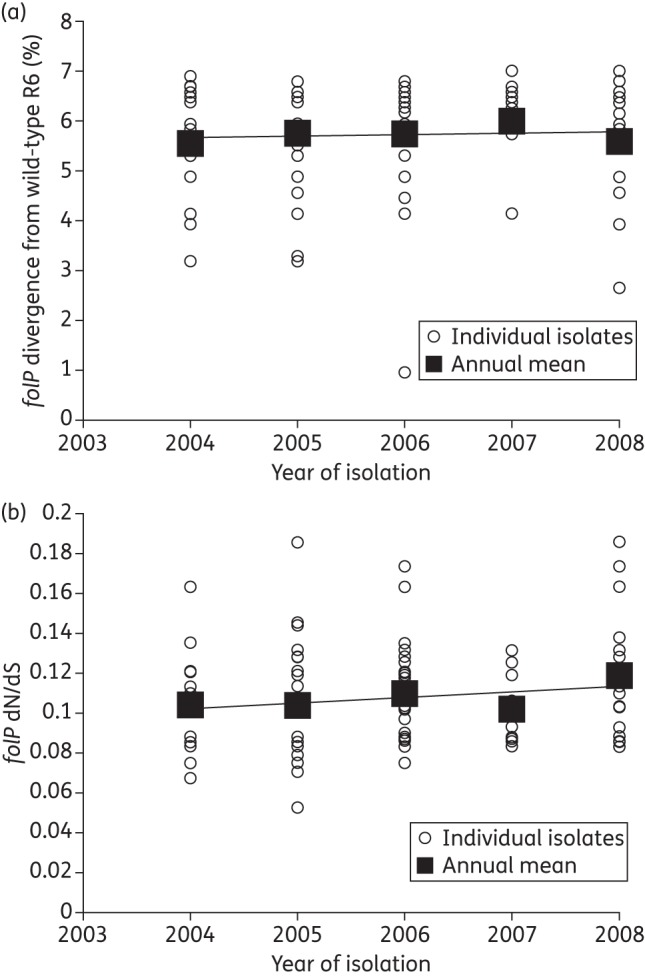
Diversity of *folP* from Malawian pneumococci, 2003–08. (a) Annual sequence divergence of *folP* from pneumococci in relation to the co-trimoxazole-susceptible laboratory reference strain *S. pneumoniae* R6. (b) Graph showing the annual dN/dS of *folP* from pneumococci in relation to the co-trimoxazole-susceptible laboratory reference strain *S. pneumoniae* R6.

The mean nucleotide sequence divergence of *folP*, in relation to *folP*_R6, was relatively stable over the isolate collection period, from 5.66% in 2004 to 5.72% in 2008 (*P* = 0.6) (Figure 1a). The mean dN/dS ratio of *folP* also remained stable over the same period, from 0.10 in 2004 to 0.12 in 2008 (*P* = 0.09) (Figure 1b).

**Figure 2.**
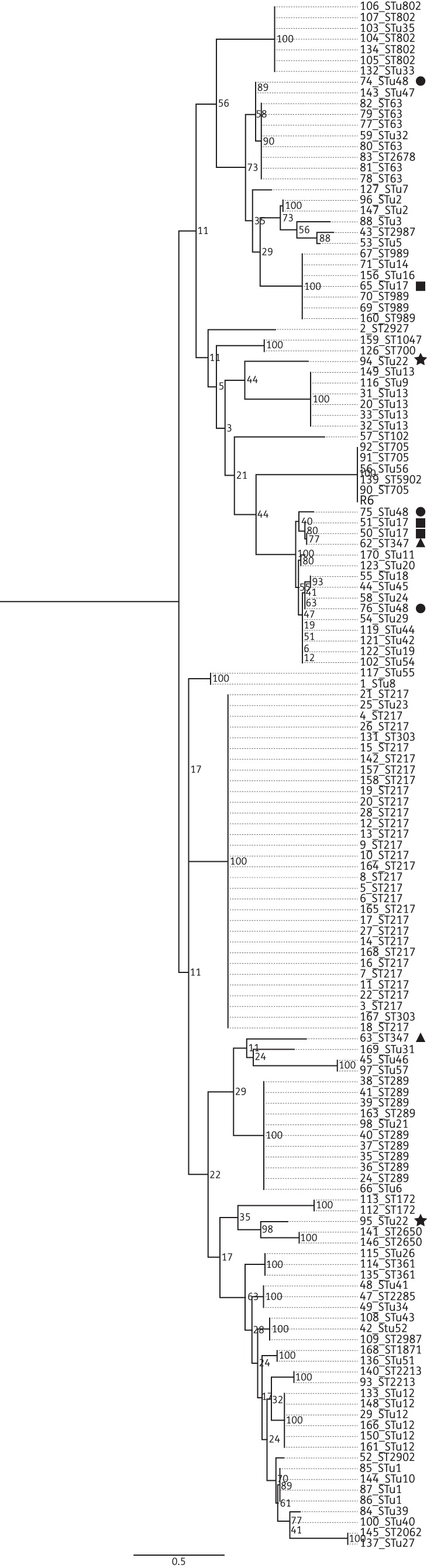
Maximum likelihood phylogenetic tree based on the *folP* SNPs from 143 Malawian pneumococci, annotated with serotype and ST. STs represented more than twice in the study population that did not cluster on the same clade are highlighted by a symbol: STunknown22, star; ST347, triangle; Stu48, square; STunknown17, circle. Bootstrap values are shown on each branch. The scale bar represents the number of SNPs.

A maximum likelihood tree constructed using the nucleotide sequence of *folP* from the 143 study isolates is presented in Figure 2. With the exception of isolate 127, all of the sulfamethoxazole-susceptible isolates formed a single clade separate from the trimethoprim-susceptible *folA*_R6. All of the serotype 1 isolates, regardless of their ST, shared an identical *folP* sequence and clustered in a distinct clade. Of the 20 STs represented more than once in the study population, 16 shared identical *folP* sequences within all isolates belonging to that ST; 16 separate clades were formed in the phylogeny, each consisting of isolates with only a single ST. The remaining four STs did not share identical *folP* sequences, and the isolates belonging to each ST sat on a distinct lineage within the phylogeny. Three serotype 7F, STunknown48 isolates were represented in the study collection (isolates 74, 75 and 76). Isolates 75 and 76 shared identical *folP* sequences and grouped together in the same clade; however, isolate 74 sat in a distinct clade with ST16, ST63, ST6872 and STunknown32. The *folP* sequence of isolate 74 was 6.8% divergent from that of isolates 75 and 76. Isolate 74 had an *folP* sequence identical to that of the four other STs belonging to the same clade.

Two of three serotype 12B STunknown17 isolates, 50 and 51, grouped in the same clade; however, a third, isolate 65, sat in a clade with serotype 12B ST989. Isolates 51 and 50 had an identical *folP* sequence, but the sequence diverged from that of the *folP* of isolate 65 by 7.2%. The two 18B, STunknown22 isolates (isolates 94 and 95) represented in the study population did not sit together on the tree. There was 5.6% sequence divergence between the *folP* from the two isolates. The two serotype 19F, ST347 isolates also sat apart in distinct clades (7.3% sequence divergence). The nucleotide sequence of *folP* from isolate 62 was unique within the study population, whilst isolate 63 had an identical *folP* sequence to isolate 12B, STunknown17, which sat on the same clade as isolate 63.

### Molecular markers of trimethoprim resistance

**Figure 3.**
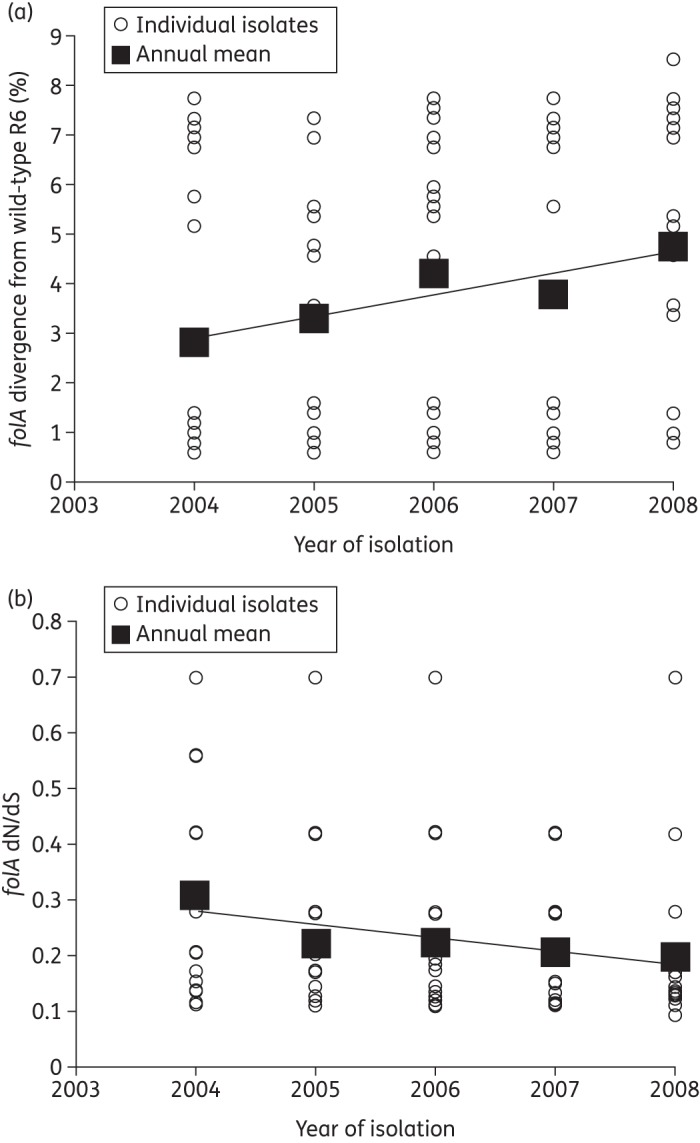
Diversity of *folA* from Malawian pneumococci 2003–08. (a) Annual sequence divergence of *folA* from Malawian pneumococci in relation to the co-trimoxazole-susceptible laboratory reference strain *S. pneumoniae* R6. (b) Graph showing the annual dN/dS of *folA* from Malawian pneumococci in relation to the co-trimoxazole-susceptible laboratory reference strain *S. pneumoniae* R6.

Nucleotide sequences of *folA* from all isolates were determined and compared with *folA*_R6. There was an annual increase in sequence divergence from 2.82% in 2004 to 4.75% in 2008; however, this increase was not significant (*P* = 0.125) (Figure 3a). The dN/dS ratio of *folA* could not be calculated for 12 isolates as these isolates only displayed non-synonymous SNPs within the gene. The dN/dS ratio was calculated for the remaining 131 isolates. There was an annual decrease in the dN/dS ratio of *folA*, from 0.3 in 2004 to 0.2 in 2008; this decrease was not significant (*P* = 0.72) (Figure 3b).

Amino acid sequences of DHFR were predicted and analysed. Amino acid substitutions at two residues were dominant within the study population, I100L and substitutions at residue 92 (Asp-92 to Ala, Gly, Val or Thr) (Table 2). The trimethoprim-resistant isolates could be assigned to one of three genotypic groups: those containing Ile-100-Leu (10%); those containing Ile-100-Leu in combination with a substitution at residue 92 (56%); and those containing a substitution at residue 92 with the wild-type amino acid at residue position 100 (34%). In this third group, the amino acid substitution at residue 92 was always Asp-92-Ala.

**Table 2.** Amino acid variation at residues 92 and 100 of DHFR from trimethoprim-resistant pneumococci in relation to the co-trimoxazole-susceptible laboratory reference *S. pneumoniae* R6

DHFR mutation	Number of isolates (%)
Asp-92-Arg, Ile-100-Leu	54 (41)
Asp-92-Arg	41 (30)
Asp-92-Thr, Ile-100-Leu	19 (14)
Asp-92-Gly, Ile-100-Leu	10 (8)
Asp-92-Val, Ile-100-Leu	8 (6)

Other amino acid substitutions that were unique to the trimethoprim binding site of the resistant isolates were Pro-70-Leu, Ala-78-Thr, Glu-94-Asp and Leu-135-Phe. Six trimethoprim-resistant isolates contained Asp-92-Ala, with no other amino acid substitutions in the trimethoprim-binding site.

**Figure 4.**
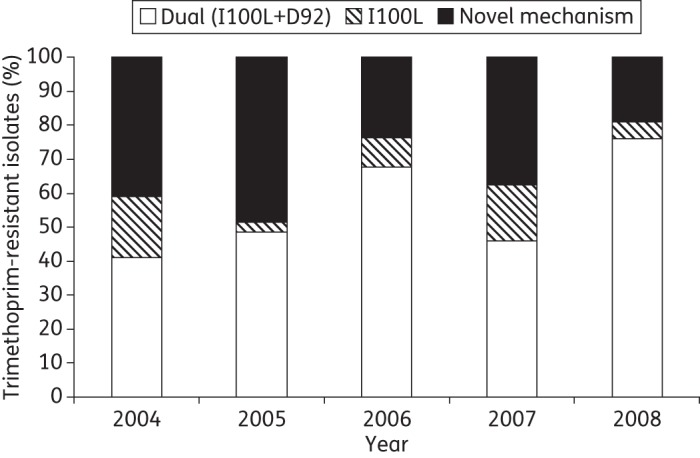
Annual distribution of three resistance genotypes amongst 137 trimethoprim-resistant Malawian pneumococci.

The distribution of three genotypic groups amongst the trimethoprim-resistant pneumococci on an annual basis is shown in Figure 4. A trend analysis comparing all of the study years showed a significant increase in the number of pneumococci possessing the dual genotype over the study period (*P* = 0.045). The change in the number of pneumococci possessing the Ile-100-Leu phenotype was not significant (*P* = 0.67). There was a decrease in the number of pneumococci with the Asp-92 mutations; however, this was not significant (*P* = 0.06).

**Figure 5.**
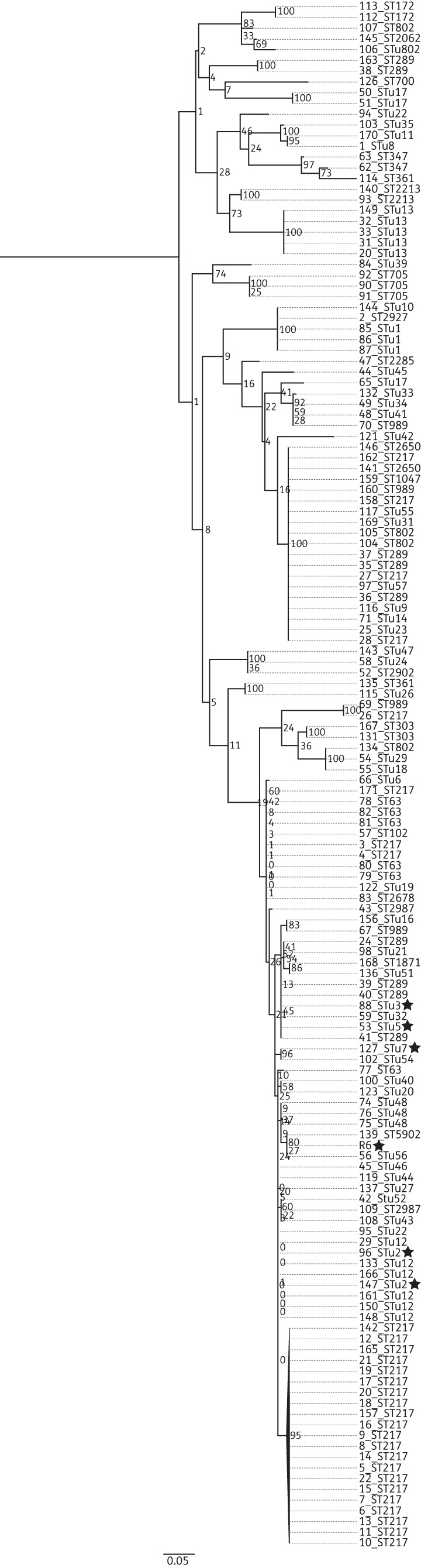
Maximum likelihood phylogenetic tree based on the *folA* SNPs from 143 Malawian pneumococci, annotated with serotype and ST. Trimethoprim-susceptible isolates are highlighted with a star. Bootstrap values are shown on each branch. The scale bar represents the number of SNPs.

A maximum likelihood tree constructed using the 138 variable sites in *folA* from the 143 study isolates is presented in Figure 5. The trimethoprim-susceptible isolates did not cluster together on the tree, sitting on four distinct clades. The ST was a poor indicator of how isolates would cluster on the tree. Only three STs (STunknown48, ST705 and STunknown13) shared identical *folA* sequences and clustered together on the tree. The remaining 17 STs did not cluster together and were dispersed across multiple *folP* lineages within the phylogeny. Isolates belonging to ST289 were represented in four distinct *fo*l*P* lineages.

## Discussion

We propose that extensive use of both co-trimoxazole and sulfadoxine/pyrimethamine in Malawi has been a major driving force behind the evolution and maintenance of co-trimoxazole resistance in Malawian pneumococci. Resistance amongst pneumococci to co-trimoxazole was already high (74%) before the introduction of CPT in 2002. A previous study of Malawian children aged <5 years found that sulfadoxine/pyrimethamine treatment led to a significant increase in the proportion of children colonized with co-trimoxazole-non-susceptible pneumococci.^23^ After the introduction of CPT, resistance rose to 92%. It has previously been reported that there is a significant correlation between regional consumption of co-trimoxazole and resistance in pneumococci in the following year^24^ and we believe the increased resistance is a result of extensive use of co-trimoxazole locally.

All of the sulfamethoxazole-resistant isolates possessed a 3 or 6 bp insertion in the nucleotide sequence encoding amino acids 58–67 of DHPS. This has previously been described as a hot spot for mutations in sulfamethoxazole-resistant pneumococci.^3,9,10^ The most common insertions led to the duplication of Arg-58 and Pro-59 and the insertion of an arginine residue between Gly-60 and Ser-61. These mutations have been previously shown to confirm resistance.^8^ Two mutations, the Val_64_Glu_65_ and Gly_60_Thr_61_ repetitions described here, are novel. The role of these mutations in sulfamethoxazole resistance has yet to be established; however, their location in the sulphonamide-binding site amongst resistant isolates strongly suggests that these mutations reduce the affinity of sulfamethoxazole for DHPS.

The phenotypic and genotypic characterization presented here shows that Malawian pneumococci are almost universally resistant to sulfamethoxazole. Previous studies have shown that insertions in the sulphonamide-binding site leading to a drastic reduction in the binding affinity to sulfamethoxazole have minimal impact on the binding affinity of DHPS to its natural substrate.^10^ This suggests that, despite removal of sulfadoxine/pyrimethamine selective pressure on *folP*, sulfamethoxazole-resistant pneumococci will continue to dominate within the population as the resistance imposes minimal fitness cost.

Clonal dissemination of specific STs appears to be responsible for the dominance of sulphonamide resistance in Malawi. Phylogenetic analysis demonstrated that ST was a strong indicator of isolate grouping. The 6.8% sequence divergence between *fol*P from isolate 74 and *fol*P from 75 and 76, all of which were STunknown48, is unlikely to have resulted from spontaneous mutation alone. The *folP* sequence encoded by isolate 74 was identical to that of isolates from four different serotypes, all of which were in the same clade. The presence of multiple STs in the same *folP* lineage is strongly suggestive of recombination. The *folP* sequence of ST989 of isolate 70 was identical to that of isolate 65. The high level of sequence divergence between *folP* from isolates belonging to ST989, STunknown22 and ST347 (7.2%, 5.6% and 7.3%, respectively) suggests that *folP* encoding DHPS with reduced affinity for sulfamethoxazole has spread via recombination.

Our data show that, of the Malawian pneumococci that were trimethoprim resistant (MIC >32 mg/L), one-third possessed a previously unreported resistance genotype, a mutation at residue 92 without Ile-100-Leu in DHFR, and suggest a potentially novel resistance mechanism. Previous studies report I100L as critical for the generation of trimethoprim resistance in pneumococci, while an additional mutation at residue 92 causes a further significant increase in MIC.^6^ The substitution at residue 92 without the Ile-100-Leu mutation has been considered insufficient to confer trimethoprim resistance.^25^ The majority of isolates (56%) possessed Ile-100-Leu with an additional mutation at residue 92, and 10% contained Ile-100-Leu alone. Amongst those exhibiting the novel genotype there were no further amino acid substitutions in the trimethoprim-binding site. Amino acid substitutions outside the trimethoprim-binding site in DHFR were also found in the five trimethoprim-susceptible isolates, suggesting that they do not play a role in resistance. Trimethoprim-resistant pneumococci that do not possess the Ile-100-Leu substitution in DHFR have not been described previously. Further investigation is required to determine whether this novel pattern is unique to Malawi or has simply not yet been described in other geographical locations due to the small numbers of *folA* genes sequenced to date.

Our data suggest that extensive co-trimoxazole use has biased the selection of pneumococci resistant to trimethoprim. The number of isolates with a mutation at position 92 but no Ile-100-Leu mutation and those encoding the Ile-100-Leu with the wild-type residue at position 92 decreased between 2004 and 2008. In parallel there was a significant increase in those possessing Ile-100-Leu with an additional mutation at residue 92. Over the same time period CPT increased substantially in the population. The additional mutation at position 92 has been shown to significantly reduce the affinity of DHFR for trimethoprim in relation to the Ile-100-Leu alone.^6^

Of the 20 STs represented more than once in the study population, only three shared an identical *folA* sequence and clustered together in the phylogeny. ST did not predict how the remaining 17 STs would cluster in the phylogeny, suggesting that recombination plays a major role in the spread of trimethoprim resistance. It is unclear why *folA* and *folP* are being disseminated by different mechanisms.

There was an increase in the sequence divergence of *folA* amongst the isolates, matched by a decrease in dN/dS ratio from 2004 to 2008. Neither change was significant, suggesting the sequence divergence of *folA* from that of *folA*_R6 has remained constant. During the same time period the sequence divergence of *folP* from *folP*_R6 remained relatively stable, with minor drift. The years 2002 and 2003 were excluded from the analysis because the sample contained fewer than three isolates from each of these years. The dN/dS ratio also remained stable from 2004 to 2008 despite extensive co-trimoxazole use. Irrespective of isolation date, all resistant isolates displayed very high-level resistance to both trimethoprim and sulfamethoxazole. The sequence data therefore demonstrate that the evolution of both *folA* and *folP* is stable in the Malawian pneumococcal population. We postulate that the strong selective pressure of extensive co-trimoxazole and sulfadoxine/pyrimethamine use over more than a decade means mutations additional to *folA* and *folP* will provide no additional fitness advantage, as the bacteria are now almost universally resistant.

In conclusion, the employment of both an *in vitro* and an *in silico* analytical approach has enabled us to describe in detail the genetic basis of co-trimoxazole resistance in *S. pneumoniae* in Malawi. By creating robust phylogenies we have established that clonal dissemination and recombination are contributing to the spread of co-trimoxazole resistance. Previously reported conventional testing for co-trimoxazole resistance in countries with high HIV seroprevalence has led to considerable debate as to why, with such high levels of pneumococcal resistance, clinical efficacy is maintained.^26,27^ However, in the context of stabilizing evolution *in silico*, we suggest that the protective effects of co-trimoxazole in this vulnerable population may have changed since the relevant trials were conducted >10years ago.^28,29^ Our findings are likely to be generalizable to other countries where co-trimoxazole and sulfadoxine/pyrimethamine have been widely used. We therefore suggest that the efficacy of co-trimoxazole prophylaxis against pneumococcal infection in HIV/AIDS in sub-Saharan Africa should be re-evaluated.

## Funding

This work was supported by a Wellcome Trust Major Overseas programme award (award no. 084679/Z/08/Z). Activities at the Wellcome Trust Sanger Institute were supported by Wellcome Trust core funding (grant number 098051). The funders had no role in study design, data collection and analysis, decision to publish, or preparation of the manuscript.

## Transparency declarations

None to declare.
